# Developing targets to control multiple pest fly species: violet polyester targets effectively attract tsetse (Diptera: Glossinidae), stable flies (Diptera: Muscidae), and house flies (Diptera: Muscidae)

**DOI:** 10.1093/jee/toaf113

**Published:** 2025-06-20

**Authors:** Michael N Okal, Sheillah J Akinyi, Roger D Santer

**Affiliations:** International Centre of Insect Physiology and Ecology, Nairobi, Kenya; Africa Technical Research Center, Vector Health International, Arusha, Tanzania; International Centre of Insect Physiology and Ecology, Nairobi, Kenya; Department of Life Sciences, Aberystwyth University, Aberystwyth, Ceredigion, UK

**Keywords:** color, sleeping sickness, tiny target, trap optimization, vector control

## Abstract

Tsetse (*Glossina* spp.), stable (*Stomoxys* spp.), and house flies (*Musca domestica* L.) impact humans and animals through bites and/or disease transmission. Tsetse are controlled using insecticide-coated fabric targets that are traditionally blue and/or black, but a violet polyester has now been engineered for greater attractiveness. Here, we investigate whether violet targets are also effective against stable and house flies. We sampled flies by electrocution at 1.0 (H) × 0.5 m (W) targets in Kenya, comparing catches at violet targets, and blue and black targets made from polyesters used in commercial traps and targets. Catches of female but not male *Glossina pallidipes* (Austen) were significantly greater at violet and black than at blue. Catches of *Stomoxys calcitrans* (L.)*, S. niger niger* (Macquart), and *Musca domestica* were significantly greater at violet than at blue or black. Blue was significantly preferred over black for *Stomoxys* spp., and black over blue for *M. domesti*ca. Thus, violet targets were individually or jointly most effective against all species investigated. Catches of *Stomoxys* spp. and *M. domestica* were significantly greater at larger (1.0 × 0.5 m and 1.0 × 1.0 m) than at smaller (0.25 × 0.25 m and 0.5 × 0.5 m) violet targets. There were no significant effects of horizontal or vertical orientation for 1.0 × 0.5 m violet targets but catches of *Stomoxys* spp. tended to be greater with horizontal orientation, and catches of *M. domestica* greater with vertical orientation. Our results suggest that simple violet targets can be effective against multiple pest fly species.

## Introduction

Flies impact the health and well-being of humans and animals worldwide, but effective fly control can help reduce these impacts. Much research has justifiably been directed at optimizing devices for the efficient control of specific fly species that cause the greatest impacts (eg tsetse; Vale 1993, Lindh et al. 2009, Esterhuizen et al. 2011, Torr and Vale 2015). Such work leads to highly effective but specialized control devices, and, therefore, high diversity in the available fly control options. To simplify fly control, an alternative approach is to develop multi-purpose traps with minimal species bias (eg Mihok 2002, 2024, Mihok et al. 2006, Mihok and Carlson 2021).

Tsetse (*Glossina* spp.) range across sub-Saharan Africa, and their bites spread trypanosome parasites that cause Human African Trypanosomiasis (HAT, sleeping sickness) and Animal African Trypanosomiasis (AAT, nagana). Tsetse of the savannah species group are the main vectors of AAT and Rhodesian HAT (rHAT), a zoonosis comprising a small percentage of HAT cases. Decades of field research have determined that savannah tsetse can be controlled using large (1.0 × ca. 2.0 m) blue and/or black cotton fabric panels coated with insecticide and accompanied by odor lures (Vale 1993, Green 1994) and/or by insecticide-treating cattle (Hargrove et al. 2000, Torr et al. 2007). The vast majority (> 95 %) of HAT cases comprise Gambian HAT (gHAT), an anthroponosis spread primarily by tsetse of the riverine species group. However, due to differences in ecology and behavior, vector control of these flies using methods designed for savannah tsetse was not considered economically viable (eg Solano et al. 2013). However, it was realized that odor lures are ineffective for riverine tsetse, such as *Glossina fuscipes fuscipes*, and that catches of these flies were relatively less affected by decreases in target size compared to savannah tsetse, leading to the development of Tiny Targets (adjacent 0.25 × 0.25 m panels of insecticide-coated blue fabric and net) specifically for the more economical control of riverine tsetse (Lindh et al. 2009, Esterhuizen et al. 2011, Torr and Vale 2015). Tiny Targets are constructed from blue polyester rather than cotton due to the fabric’s greater robustness, color fastness, and ability to hold insecticides under field conditions (eg Lindh et al. 2012). Tiny Targets have allowed affordable vector control to contribute to the reduction in reported gHAT cases below 1000 per year (Franco et al. 2022).

In developing Tiny Targets, it was apparent that typical blue polyesters were less attractive to tsetse than traditional phthalogen blue cotton (Lindh et al. 2012), but the blue polyesters produced for Tiny Targets have since been refined by reducing UV reflectance, since high UV reflectance is known to negatively affect a target’s attractiveness (Green and Flint 1986, Green 1988, Lindh et al. 2012). Meanwhile, a new violet polyester fabric has been developed using fly’s-eye-view color modeling approaches to rationally design a more attractive fabric (c.f. Santer and Allen 2024). In this work, the photoreceptor signals a fly would experience when viewing a fabric were calculated and used as predictors in statistical models that explained tsetse attraction observed in field studies (Santer 2014, 2017). Attempts were then made to identify a dye mixture that would more effectively elicit the photoreceptor signals associated with attraction, resulting in a putatively more attractive violet polyester (this fabric is in fact purple to a human eye, but was named for the dye used to produce it). The violet fabric was validated in field tests where it often attracted significantly more savannah tsetse than a standard black cotton and/or a typical blue polyester using the large target configuration normally employed for those flies (Santer et al. 2019). Meanwhile, targets of the commercially produced blue polyester used in Tiny Targets and tsetse traps did not attract more tsetse than the black cotton standard (Santer et al. 2019). The violet fabric also caught more riverine tsetse in the Tiny Target configuration than typical blue polyester targets. Analyses predicted that violet would catch more such tsetse on average than the commercially produced blue fabric, though the high variability in catches at Tiny Targets meant that the performance difference between these two fabrics would likely be negligible when deployed in this format (Santer et al. 2021). Nevertheless, the violet polyester has performed well for different tsetse species and target configurations and has thus been proposed to be a better general choice for tsetse target construction (Santer et al. 2021).

The visual systems of tsetse appear to be typical of many higher flies (Hardie 1986, Hardie et al. 1989, International Glossina Genome Initiative 2014, Attardo et al. 2019), and there may also be cross-species similarities in the visual control of behaviors that attract flies to traps and targets (Santer et al. 2023). Therefore, the extensive research into tsetse targets might be translated into control devices for other species, including stable flies and house flies. This idea is supported by the fact that successful multi-species traps have already been developed, though their design is more complex than the Tiny Target (c.f. Mihok 2002, 2024, Mihok et al. 2006, Mihok and Carlson 2021). Stable flies inflict painful bites and are mechanical vectors of several pathogens, making them among the most damaging insect pests of dairy cattle worldwide (Rochon et al. 2021). The US cattle industry alone has been estimated to lose US$2,211 million/year due to these flies (Taylor et al. 2012). Blue/black Nzi traps captured tsetse of various species while also capturing stable flies (Mihok 2002, Mihok et al. 2006), and traditional black and blue cotton/polyester tsetse targets have also proven effective against stable flies in the United States (Hogsette and Foil 2018), demonstrating that common principles likely guide the behavior of these flies. Similar to work on tsetse, studies on stable flies have recently explored alternative blue polyester fabrics to use in place of cotton, identifying a particular blue polyester for further development (Onju et al. 2020), and blue sticky traps have been explored as a cheaper alternative to fabric targets for these flies (Sharif et al. 2020). However, contrasting this work, red fabrics have recently been reported to more selectively attract stable flies in sticky trap and Vavoua trap configurations (Getahun et al. 2024). House flies are responsible for the mechanical transmission of many pathogenic organisms and corresponding impacts on human and animal health, and economic losses due to these flies in the United States alone are estimated at US$1 billion/year (Geden et al. 2021, Jones et al. 2024). The color (blue, green, yellow, or white) of a small sticky trap had no effects on house fly catches in a poultry unit (Hanley et al. 2009), but given a choice of lights, house flies were attracted by blue and white but repelled by yellow (Diclaro et al. 2012), suggesting possible parallels with the color-guided behavior of other flies. In addition to providing effective control devices for these flies, targets effective against multiple species might aid in the longer-term suppression of tsetse populations, since the additional benefits provided by a target in controlling nuisance species may safeguard its long-term deployment once tsetse are infrequently encountered and cost:benefit ratios in that context are shifted.

This study focuses on the effectiveness of previously developed tsetse control targets against stable and house flies. We first tested the optimized violet polyester fabric developed to attract tsetse more effectively (Santer et al. 2019) and the blue and black polyester fabrics commercially produced for tsetse traps and targets by Vestergaard S.A. under the “ZeroFly” product name, sampling flies by electrocution over 1.0 (H) × 0.5 m (W) fabric panels. Finding violet to be effective for all species, we next tested the effects of altering a violet target’s size from 0.25 × 0.25 m up to 1.0 × 1.0 m. Finally, we investigated the effect of vertical or horizontal orientation for 1.0 × 0.5 m violet targets.

## Materials and Methods

### Targets

**Fig. 1.**
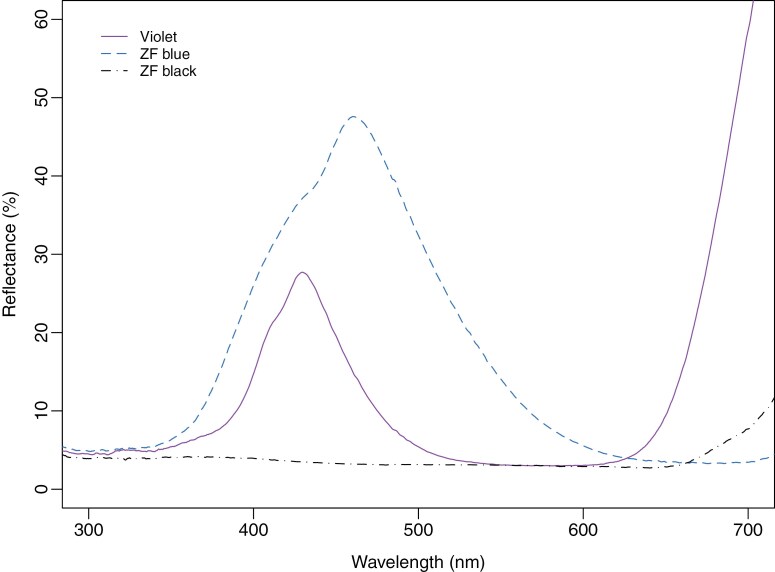
Reflectance spectra of fabrics tested in this work. Spectra were measured using an Ocean Optics USB4000 spectrometer, PX-2 pulsed xenon light source flashing with a 30 ms period, reflectance probe, and WS-1-SL standard (Ocean Insight Inc., Orlando, FL, USA). The reflectance probe was angled 45° to vertical, and its tip was positioned at 6 mm from the fabric. A 120 ms integration period, boxcar width of 5, and 25 scans to average were used. Plots are the average of three replicate spectra for each of two reflectance probe azimuth angles on one side of the fabric.

Targets comprised polyester fabric panels ranging from 0.25 × 0.25 m to 1.0 × 1.0 m in size. We tested three different fabrics in total. These comprised (i) blue and (ii) black fabrics produced by Vestergaard S.A. for its ZeroFly tsetse traps and targets (henceforth “ZF blue” and “ZF black”), and (iii) a violet fabric (purple to a human eye) designed for increased attractiveness to tsetse (Toray Textiles Europe Ltd., Mansfield, UK; Santer et al. 2019, 2021) (Fig. 1). Flies visiting a target were sampled using an overlying grid of electrocuting wires, but additional grids flanking a target were not employed.

### Data Collection

#### Evaluating the Relative Attractiveness of Colored Fabrics

To investigate the variation in fly catches between 1.0 (H) × 0.5 m (W) targets made from either ZF blue, ZF black or violet polyester fabrics, we conducted an experiment in the human-wildlife-livestock interface of the Shimba Hills National Reserve (SHNR) in Kwale County, Kenya (Latitude −4.1742°S and Longitude 39.4602°E; Altitude 250 m) in March 2021, in which we predominantly caught *Glossina pallidipes* (Austen) and *Musca domestica* (L.). This comprised 10 d of sampling in the morning (09:00 to 12:00 h), with three targets and three different sites tested on any given day and targets rotated between sites on subsequent sampling days. Sample sites were relocated on sampling day 8, meaning 6 sites were tested in total. We conducted a similar experiment in Amboseli National Park (ANP), in Kajiado County, Kenya (Latitude −2.7013 and Longitude 37.3197; Altitude 1180m) in June 2021, in which we predominantly caught *Stomoxys calcitrans* (L.) and *S. niger niger* (Macquart). This comprised 9 sampling days, with three targets and three different sites tested on any given day, and targets rotated between sites on subsequent sampling days. We sampled flies during separate morning (09:00 to 12:00 h) and afternoon (12:00 to 15:00 h) sampling periods.

#### Evaluating the Effect of Target Size and Orientation

In June 2021, we investigated variation in catches between violet targets of 0.25 × 0.25 m, 0.5 × 0.5 m, 1.0 (H) × 0.5 m (W), and 1.0 × 1.0 m in ANP. The experiment comprised 16 d of sampling, with four targets and four different sites tested on any given day, and targets rotated between sites on subsequent sampling days. We also investigated variation in catches between 1.0 × 0.5 m violet targets in either vertical or horizontal orientation in ANP. This experiment comprised 8 d of sampling, with two targets and two different sites tested on any given day, and targets rotated between sites on subsequent sampling days. We sampled flies during separate morning (09:00 to 12:00 h) and afternoon (12:00 to 15:00 h) sampling periods, and predominantly caught *M. domestica* and *Stomoxys* spp.

Data are provided as Supplementary File S1.

### Statistical Methods

We analyzed the total numbers of *G. pallidipes* (separated by sex), *M. domestica*, *S. calcitrans,* and *S. niger niger* caught at each sampling period. Fly catches were analyzed using Generalized Linear Mixed Models (GLMM) implemented using glmmTMB (Brooks et al. 2017) and R version 4.4.2. We included a fixed effect of target type, and random intercepts for sampling day, sampling site, and sampling time for the experiments where it was relevant. We specified a negative binomial (nbinom2) distribution and log link function, and checked model fits using DHARMa (Hartig 2024). In two cases (*M. domestica* catches across fabric types, and *S. niger niger* catches across target sizes), a possible fitting issue was identified, but since a quasi-Poisson (nbinom1) model supported an identical conclusion and had no such issues, we report the initial model for simplicity. Posthoc tests were LSD tests implemented using the emmeans package (Lenth 2024).

### Ethics

Experiments were conducted on invertebrate animals (pest flies) and were not subject to regulation. However, experiments were declared to the Animal Welfare and Ethics Review Board, Aberystwyth University, and aligned with UK research ethics standards. Protocols used for the study complied with the guidelines of *icipe*’s Institutional Animal Care and Use Committee.

## Results

### The Relative Attractiveness of Colored Fabrics

**Fig. 2.**
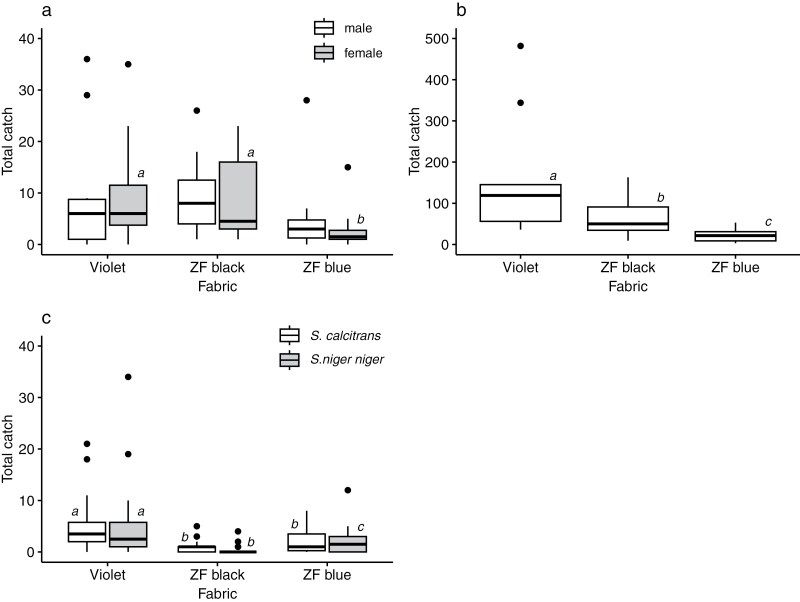
Fly catches at targets made from three different fabrics. Catches are shown for (a) *Glossina pallidipes*, (b) *Musca domestica*, and (c) *Stomoxys* spp. Boxes indicate the 25^th^, 50^th^, and 75^th^ percentiles; whiskers extend to the most extreme value, or to 1.5 x IQR with more extreme values plotted as points. Boxes within a given sex or species that do not share a letter are significantly different at *P* < 0.05 according to unadjusted posthoc tests.

We initially compared the attraction of flies to violet, ZF blue, and ZF black fabrics in 1.0 (H) × 0.5 m (W) targets. We did this through experiments near SHNR, where we caught large numbers of *Glossina pallidipes* and *Musca domestica*, and experiments in ANP, where we caught large numbers of *Stomoxys calcitrans* and *S. niger niger* (Fig. 2).

For *G. pallidipes* at SHNP (Fig. 2a), catches of females differed significantly across the three fabrics (negative binomial GLMM, Wald Χ^2^_2_ = 22.173, *P* < 0.001), but the trend for males was marginally non-significant (negative binomial GLMM, Wald Χ^2^_2_ = 5.068, *P* = 0.079). For females, catches at the violet and ZF black targets did not differ significantly but were significantly greater than those at the ZF blue target (LSD tests, *P* < 0.05).

Patterns for *M. domestica* at the same location were broadly similar (Fig. 2b). Catches differed significantly across the three fabrics (negative binomial GLMM, Wald Χ^2^_2_ = 66.315, *P* < 0.001), but in this case, the catch at the violet target significantly exceeded that at ZF black, which significantly exceeded that at ZF blue (LSD tests, *P* < 0.05).

Catches of *Stomoxys* spp. at ANP presented a different pattern, wherein the ZF black target was markedly less attractive (Fig. 2c). Catches differed significantly across fabrics for *S. calcitrans* (negative binomial GLMM, Wald Χ^2^_2_ = 20.951, *P* < 0.001), and for *S. niger niger* (negative binomial GLMM, Wald Χ^2^_2_ = 25.022, *P* < 0.001). For both species, catches at the violet target significantly exceeded those at ZF blue and ZF black. The catch at ZF blue significantly exceeded that at ZF black for *S. niger niger* but not for *S. calcitrans* (LSD tests, *P* < 0.05).

### Effects of Target Size and Orientation

**Fig. 3.**
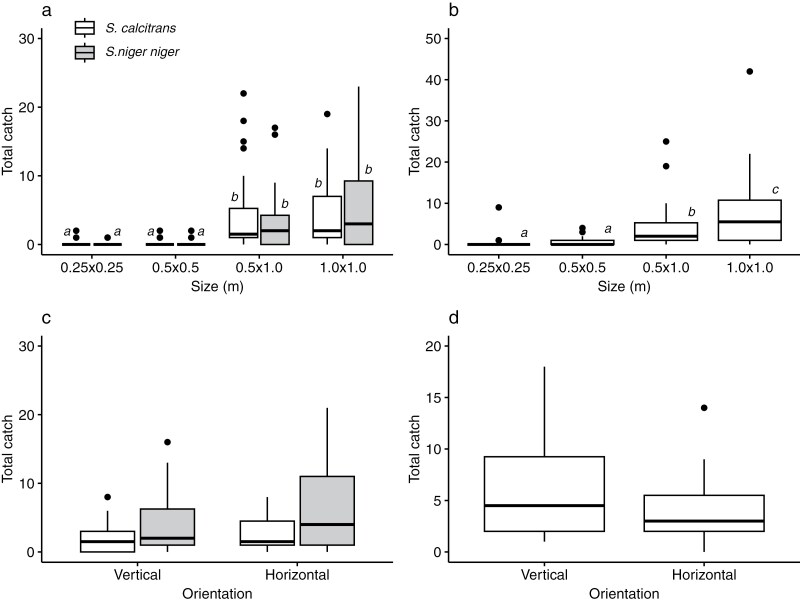
Fly catches at violet targets with different sizes (a, b) and orientations (c, d). Catches are shown for *Stomoxys* spp. (a, c), and *Musca domestica* (b, d). Boxes indicate the 25^th^, 50^th^, and 75^th^ percentiles; whiskers extend to the most extreme value, or to 1.5 * IQR with more extreme values plotted as points. Boxes within a given species that do not share a letter are significantly different at *P* < 0.05 according to unadjusted posthoc tests.

Since violet targets were effective for all three species groups, we next investigated the effectiveness of violet targets with different sizes and orientations in two experiments in ANP that caught both *M. domestica* and *Stomoxys* spp. (Fig. 3).

Catches of both *M. domestica* and *Stomoxys* spp. tended to increase with target size (Fig. 3a,b). There were significant differences in catches across targets of different sizes for *S. calcitrans* (negative binomial GLMM, Wald Χ^2^_3_ = 72.979, *P* < 0.001), *S. niger niger* (negative binomial GLMM, Wald Χ^2^_3_ = 64.997, *P* < 0.001), and *M. domestica* spp. (negative binomial GLMM, Wald Χ^2^_3_ = 93.055, *P* < 0.001). In all cases, catches at the smallest two targets did not differ, and catches at the largest two targets significantly exceeded those at the smaller targets. There was no significant difference in catches at the largest two targets for *Stomoxys* spp., but for *M. domestica* the larger of the two targets had a significantly greater catch (LSD tests, *P* < 0.05).

Focusing on rectangular 0.5 × 1.0 m violet targets, more *M. domestica* tended to be caught at vertically oriented targets, and more *Stomoxys* spp. at horizontally oriented targets (Fig. 3c,d). However, the difference in catches was not significant for *S. calcitrans* (negative binomial GLMM, Wald Χ^2^_1_ = 0.773, *P* = 0.379), *S. niger niger* (negative binomial GLMM, Wald Χ^2^_1_ = 1.973, *P* = 0.160), or *M. domestica* (negative binomial GLMM, Wald Χ^2^_1_ = 1.905, *P* = 0.168).

## Discussion

In this study, we investigated the effectiveness of fabric targets originally developed for tsetse control against several different pest fly species. We found that 1.0 × 0.5 m violet and ZF black targets caught similar numbers of *G. pallidipes* of both sexes, but catches of females were significantly greater at these targets than at ZF blue targets. However, violet targets caught significantly more *M. domestica* and *Stomoxys* spp. than ZF black or ZF blue targets did, with the relative effectiveness of ZF black and ZF blue targets differing between species. Thus, compared to currently produced fly control fabrics, violet targets were individually or jointly most effective for all species investigated. We also compared the numbers of flies caught at violet targets of different sizes and orientations, finding that larger targets caught significantly more *M. domestica* and *Stomoxys* spp. than smaller targets, but that catches at 1.0 × 1.0 m and 1.0 × 0.5 m targets were similar for *Stomoxys* spp. The orientation of a 1.0 × 0.5 m target had differing, non-significant effects on the numbers of *M. domestica* and *Stomoxys* spp. caught. Thus, we conclude that a 1.0 × 0.5 m or larger violet target would provide a simple and effective tool for controlling multiple pest fly species.

Biting flies of many species are known to be attracted to blue or black targets (Gibson and Torr 1999, Santer et al. 2023), so these have become standard options for control devices (Green 1994). However, the violet fabric tested in this work was initially developed to more effectively attract tsetse, based on models that related calculated fly photoreceptor signals to attraction toward targets (Santer 2014, 2017, Santer et al. 2019). During previous field tests, catches of female *G. pallidipes* and female *G. m. morsitans* at violet targets exceeded those at black cotton targets in the presence or absence of an accompanying odor lure (Santer et al. 2019). Catches at violet targets also exceeded those at typical blue polyester targets for *G. m. morsitans* in the presence of an odor lure, and for female *G. m. morsitans* and female *G. pallidipes* in the absence of an odor lure (Santer et al. 2019). In that work, catches at violet and ZF blue targets were not directly compared, but since catches at ZF blue targets did not exceed those at black cotton, it was hypothesized that violet targets would be more effective than ZF blue (Santer et al. 2019). The current work supports that hypothesis by directly comparing catches of *G. pallidipes* at these targets and finding that catches of females at violet significantly exceeded those at ZF blue. A direct comparison of violet versus ZF blue targets has also been made for the riverine species *G. f. fuscipes* using the Tiny Target configuration employed for those tsetse, wherein it was concluded that the average catch at a violet target in future deployments was very likely to exceed that at a ZF blue one, but that the high variability in catches at such targets meant that the two materials were likely to perform similarly in practice (Santer et al. 2021). Thus, there is growing evidence that violet is an attractive fabric for several species of tsetse that would be effective at several different target sizes.

One contrast with previous work on tsetse is that catches for female *G. pallidipes* at violet targets did not significantly exceed those at ZF black, whilst catches at violet targets exceeded those at black cotton in previous work (Santer et al. 2019). Methodological differences between the studies might explain this. Firstly, our current work used ZF black polyester rather than black cotton. The reflectance spectra of those black fabrics differ subtly in that black cotton had a small peak in UV reflectance, and also faded during field deployment (Santer et al. 2019), so it is conceivable that ZF black fabric may be more attractive to flies, although no direct comparison has yet been carried out. Secondly, the earlier work employed surface and flanking nets to sample flies landing on or circling around the target (Santer et al. 2019), whilst our current work used only surface nets. Tsetse are frequently reported to have a higher tendency to land on black versus blue fabrics, making the former highly efficient (Vale 1993). Thus, an advantage for ZF black over violet in inducing flies to land rather than circle might have masked the greater attractiveness of the violet fabric. Finally, savannah tsetse catches increase with target size due both to greater attraction and greater propensity to land (Vale 1993, Torr and Vale 2015), but the targets tested in this work were half the size of those tested by Santer et al. (2019). Thus, catches were expected to be less, and differences between catches may have been less apparent as a result.

This work aimed to determine whether violet fabric targets would also be effective against stable and houseflies. During previous field tests, catches of biting muscoids at violet targets exceeded those at black cotton targets in the presence or absence of an accompanying odor lure, and for non-biting muscoids only in the presence of such a lure (Santer et al. 2019). Catches at violet targets also exceeded those at typical blue polyester targets for biting muscoids in the absence of an odor lure (Santer et al. 2019). Meanwhile, as for tsetse, catches at ZF blue targets did not differ from those at black cotton targets for any fly group analyzed (Santer et al. 2019). Herein we found a stronger preference for violet fabric among stable and house flies than for tsetse themselves, finding that catches were significantly greater at the violet fabric than either ZF blue or ZF black, both for *Stomoxys* spp. and *M. domesti*ca. This likely indicates that similar principles drive the visually-guided behaviour of these flies and that principles gleaned from work on tsetse are transferable (c.f. Mihok 2002, Mihok et al. 2006, Hogsette and Foil 2018). If this is indeed the case, the violet polyester fabric may be a better choice than the blue polyesters recently tested against *Stomoxys* in Thailand, since all of those blue fabrics had relatively high UV reflectance (Onju et al. 2020), which is not a feature of most natural spectra and is known to reduce the attractiveness of a target to tsetse (Green and Flint 1986, Green 1988, Lindh et al. 2012). Among the ZF fabrics, blue was relatively more preferred by *S. niger niger*, no significant preference was shown by *S. calcitrans*, and black was preferred by house flies. This was a surprising result given that black targets caught more stable flies than blue ones in previous work, albeit with large but not small targets, and using different fabrics that undoubtedly would have differed in their reflectance to those tested in the current work (Hogsette and Foil 2018).

In a recent study, there were no significant differences between catches of *Stomoxys* spp. and *M. domestica* at red polyester and ZF blue sticky and Vavoua traps, and the red polyester was considered more selective for *Stomoxys* over other non-target species (Getahun et al. 2024). The special attractiveness of this red polyester is unexpected given that its reflectance begins to increase at > 600 nm, normally considered beyond the visual range of calyptrate flies based upon intracellular recordings from single photoreceptors (Hardie 1986). Thus, one possibility is that the red fabric may have been effective because it appeared to flies as a low reflectance black. However, whilst stable flies possess the same opsin types as calyptrate flies that have been studied with intracellular electrophysiology (Olafson et al. 2021), ERG recordings have reported a peak in visual sensitivity at > 600 nm (Zhu et al. 2016), and such recordings from *Drosophila* show that the peak sensitivity of the long wavelength R8y photoreceptor is considerably broader and longer than might have been expected due to a combination of screening effects and high-intensity, wide-field illumination (Sharkey et al. 2020). Thus, the possibility that stable flies can perceive the long wavelength reflectance of the red fabric cannot be ruled out. If the long wavelength reflectance of this red fabric did contribute to its attractiveness, it is intriguing to note that the violet fabric tested here also has a steep increase in reflectance at > 600 nm that has so far been considered inconsequential to its attractiveness.

Catches of tsetse are known to increase with target size, and large solid fabric panels have become standard for savannah tsetse (Vale 1993, Green 1994). However, because catches of riverine tsetse are relatively less affected by target size than savannah tsetse, smaller targets can more efficiently catch those flies per given area of fabric (Torr and Vale 2015). Because the size of a target affects both the attraction and propensity to land of tsetse, Tiny Targets also include an adjacent net panel designed to intercept circling flies and offset the reduced tendency for these flies to land on small fabric panels (Lindh et al. 2009, Esterhuizen et al. 2011). We did not re-test the known effects of target size on catches of *G. pallidipes* in the current work, but did investigate them for *M. domestica* and *Stomoxys* spp. Similar to savannah tsetse, we found that larger targets caught considerably more flies than smaller ones and that 0.25 × 0.25 m and 0.5 × 0.5 m targets caught very few flies. Although 1.0 × 1.0 m targets did catch more flies than 1.0 × 0.5 m targets, the difference in catches was not significant for *Stomoxys* spp. Similar to this result, there was no significant difference in stable fly catches at a 1 m^2^ target versus the same target rolled into a cylinder and thus presenting a taller and narrower silhouette, and no difference between catches at such targets and smaller ones ca. 60 cm in height (Hogsette and Foil 2018). It is important to note that our targets did not include flanking nets, so we cannot determine whether low catches at small targets were due to flies not being attracted to those targets, or due to them being attracted but not landing (c.f. Lindh et al. 2009, Esterhuizen et al. 2011). Consequently, we are also unable to comment on the likely effectiveness of a Tiny Target with a flanking net in capturing these flies. However, we can conclude that both house and stable flies will be effectively caught by solid violet targets in the large sizes employed for savannah tsetse control. If tsetse control were not a priority, a 1.0 × 0.5 m target would likely be more economical to control *Stomoxys* spp. alone.

We found differing effects for the orientation of a 1.0 × 0.5 m target depending on the species investigated. Catches of *Stomoxys* spp. tended to be greater for horizontally oriented targets, and catches of *M. domestica* tended to be greater at vertically oriented targets, although catches were not significantly different for either group. In previous work, *S. calcitrans* were more frequently caught at low (30 cm) than high (121 cm) sticky traps, and tall sticky traps caught flies with a distribution that had its maximum density around 20 cm above the height of the surrounding vegetation (Beresford and Sutcliffe 2008). Similar has also been observed using blue sticky traps (Sharif et al. 2020). Thus, the greater target area at the low height of a horizontally oriented target should be expected to more effectively target these flies. The fact that opposite trends were apparent for *M. domestica* may indicate a difference in flight behavior, though additional work of the kind conducted on stable flies will be needed to test this hypothesis. Thus, the precise orientation of such a target may not be critical, though it could be used to fine-tune deployments where a particular species is problematic.

Our purpose in this work was to develop recommendations for fly control devices that could be used to target multiple pest fly species simultaneously. We find that the violet polyester fabric initially developed to catch tsetse is not only more attractive to tsetse but also more attractive to *M. domestica* and *Stomoxys* spp. than commercially produced fabrics, and thus, violet provides a general-purpose fabric that is effective in the control of multiple fly species using multiple target sizes. Assuming that this fabric is deployed as a simple target without a flanking net, a larger size is preferred to sample these species efficiently, and 1.0 × 0.5 m would likely be adequate if the requirement were to control these species alone. Finally, the orientation of such a target appears not to be critical but may be chosen depending on whether *M. domestica* or *Stomoxys* spp. are the greater concern.

## Supplementary material

Supplementary material is available at *Journal of Economic Entomology* online.

toaf113_suppl_Supplementary_File_S1
